# How to Reveal Magnitude of Gene Signals: Hierarchical Hypergeometric Complementary Cumulative Distribution Function

**DOI:** 10.1177/1176934318797352

**Published:** 2018-10-18

**Authors:** Bongsong Kim

**Affiliations:** Noble Research Institute LLC, Ardmore, OK, USA

**Keywords:** Genome-wide association study, hierarchical association coefficient algorithm, hierarchical binary categorization, hypergeometric complementary cumulative distribution function, magnitude of gene signals, quantitative trait loci

## Abstract

This article introduces a new method for genome-wide association study (GWAS), *hierarchical hypergeometric complementary cumulative distribution function* (HH-CCDF). Existing GWAS methods, e.g. the linear model and *hierarchical association coefficient algorithm*, calculate the association between a marker variable and a phenotypic variable. The ideal GWAS practice is to calculate the association between a marker variable and a gene-signal variable. If the gene-signal variable and phenotypic variable are imperfectly proportional, the existing methods do not properly reveal the magnitude of the association between the gene-signal variable and marker variable. The HH-CCDF mitigates the impact of the imperfect proportionality between the phenotypic variable and gene-signal variable and thus better reveals the magnitude of gene signals. The HH-CCDF will provide new insights into GWAS approaches from the viewpoint of revealing the magnitude of gene signals.

## Introduction

Let us define gene-derived effects contributing to phenotypic observations as gene signals. Considering that a genome-wide association study (GWAS) aims to identify markers closely linked to genes controlling a trait of interest, the ideal GWAS method is to calculate the association between a marker variable and a gene-signal variable. Existing GWAS methods, e.g. the linear model and *hierarchical association coefficient algorithm* (HA-coefficient algorithm), calculate the association between a marker variable and a phenotypic variable.^1–5^ If the gene-signal variable and phenotypic variable are proportional, the existing methods will reveal the association between the gene-signal variable and marker variable. In reality, however, the gene-signal variable and phenotypic variable are not perfectly proportional, which necessarily reduces the ability of the existing methods to reveal the association between the marker variable and gene-signal variable. The *hierarchical hypergeometric complementary cumulative distribution function* (HH-CCDF) that I introduce in this article addresses the problem. The HH-CCDF is a multinomial version of *hypergeometric complementary cumulative distribution function* (H-CCDF), for which the H-CCDF is combined with *hierarchical binary categorization*.^5^ The HH-CCDF fundamentally differs from the existing methods. For example, suppose that 10 natural numbers (1 to 10) are equally split into 2 categories; 5 numbers with a greater average are assigned to category 1; and the remaining numbers are assigned to category 2. To measure the distance between categories 1 and 2, the HH-CCDF considers how many numbers out of the top 5 numbers (6 to 10) are included in category 1, whereas the existing methods consider the difference between the averages of categories 1 and 2. A great number and a small number equally contribute to the count of numbers in a category. In this regard, the count-based operation makes the HH-CCDF robust in measuring the distances between categories. Conceptualizing that this robustness must be a key to capturing the magnitude of gene signals, the HH-CCDF was developed. A simulation shown in this article demonstrates that the HH-CCDF properly reveals the magnitude of gene signals beyond simply calculating the marker-phenotype associations.

## Materials and Methods

### Example data set

Table 1 is an example data set in which the phenotypic variable is quantitative, and the marker variable is categorical.

**Table 1.** Example data set that shows a marker variable and a phenotypic variable.

Individuals	Marker variable	Phenotypic variable
ID 1	1	140
ID 2	1	122
ID 3	2	116
ID 4	1	114
ID 5	1	112
ID 6	2	108
ID 7	2	108
ID 8	0	105
ID 9	1	105
ID 10	1	102
ID 11	0	101
ID 12	0	99
ID 13	0	98
ID 14	2	97
ID 15	0	95
ID 16	1	94
ID 17	2	91
ID 18	2	86
ID 19	1	84
ID 20	0	76

### Hierarchical hypergeometric complementary cumulative distribution function

The HH-CCDF is established based on the following 3 definitions introduced in the previous study^5^:

*Definition 1.* “Hierarchical” means that all categories are stratified in ascending order based on the average of each category.
*Definition 2.* Suppose that all categorical boundaries in hierarchical stratification are fixed and observations are permutable. “Top categorization” means a condition in which observations are arranged in ascending order in each category leading to ascending order across all categories.
*Definition 3.* Suppose that *n* categories are stratified in ascending order based on the average of each category from left to right, in which *n* is the number of all categories. There are *n* − 1 categorical boundaries. At each categorical boundary, we can make 2 categories by collapsing the other categorical boundaries. Let us call the result “*hierarchical binary categorization*” and designate the right subset as *s1* and the left subset as *s2* at any categorical boundary. The *s1* is a representative subset for a respective *hierarchical binary categorization*.

The H-CCDF is as follows:

(1) $$P\;value=1-\sum_{k=0}^{d}\frac{\left(\begin{array}{c} K\\ k \end{array}\right)\left(\begin{array}{c} N-K\\ d-k \end{array}\right)}{\left(\begin{array}{c} N\\ d \end{array}\right)}$$

where *N* is the population size; *K* is the total possible successes in the population; *d* is the number of draws; and *k* is the number of observed successes.

The H-CCDF is only applicable to binary data in which an outcome from a random draw can be viewed as either a success or a failure. In Table 1, the marker variable includes 3 types of categories. Therefore, the H-CCDF is not directly applicable to Table 1. In the HH-CCDF, a categorization with *n* categories can be converted into binary categorizations in *n* − 1 ways based on the *hierarchical binary categorization*. Each binary categorization can be applied to the H-CCDF, from which *P* values of *n* − 1 can be obtained. The ultimate *P* value can be obtained by calculating the geometric mean of *P* values of *n* − 1. The HH- CCDF is as follows:

(2) $$P\;value=\sqrt[{n-1}]{\prod_{x=1}^{n-1}\left(1-\sum_{k_{x}=0}^{d_{x}}\frac{\left(\begin{array}{c} K_{x}\\ k_{x} \end{array}\right)\left(\begin{array}{c} N-K_{x}\\ d_{x}-k_{x} \end{array}\right)}{\left(\begin{array}{c} N\\ d_{x} \end{array}\right)}\right)}$$

where *n* is the total number of types of categories; *x* is the loop variable; *d_x_* is the size of the *s1* given the *x*th categorical boundary; *K_x_* has the same value as *d_x_*; *k_x_* is the size of the intersection between the *s1* in the top categorization and the *s1* in the observed categorization given the *x*th categorical boundary; and *N* is the population size.

### Simulation method

To generate a simulated data set, the previous simulation method^5^ was slightly modified. The form of the simulated data set is shown in Figure 3. The simulation procedures are as follows:

1. Create a genotypic table consisting of 500 individuals (rows) and 450 markers (columns), which is filled with 0, 1, or 2 at random.
2. Make 3 pairs of symmetrical triangles on the top and bottom edges, in which the heights of the left, middle, and right triangles are 20, 30, and 40, respectively, and the widths are all 150.
3. Replace the values in the 3 top triangles with 0s and the values in the 3 bottom triangles with 2s.
4. Generate a phenotypic vector starting at 1 in which the phenotypic observations increase by 1 to the lower tip of the upper-right triangle on the top edge (slope 1); the phenotypic observations plateau between the tips of the 2 symmetrical triangles on the right (plateau); and the phenotypic observations increase by 1 from the upper tip of the lower-right triangle on the bottom edge to the bottom edge (slope 2).
5. Calculate the associations between the marker variables and phenotypic variable.
6. Repeat 100 times Steps 1 through 5, and average the 100 results.

### Methods to compare with the HH-CCDF

To examine the HH-CCDF, I applied the HH-CCDF, *F* test (as a linear model), and HA-coefficient algorithm to the same simulated data set (Figure 3) and compared the results. The formulas for the *F* test and HA-coefficient algorithm are as follows:

(3) $$y_{ij}=\mu \;+\alpha_{i}+\varepsilon_{ij}$$

where *μ* is the mean of all observations; $\alpha_{i}$ is the constant for *i*th category as random deviation from the *μ*; and $\varepsilon_{ij}$ is the random effect containing all uncontrolled sources of variability.^6^

The HA-coefficient algorithm^5^ is as follows:

(4) $$HA=\sqrt[{n-1}]{\prod_{k=1}^{n-1}\frac{{\left[y\ln\left(x\right)-x\right]}_{btm_{\left[k\right]}}^{obs_{\left[k\right]}}}{{\left[y\ln\left(x\right)-x\right]}_{btm_{\left[k\right]}}^{top_{\left[k\right]}}}}$$

where HA is the hierarchical association coefficient; *n* is the total number of categories; *k* is the loop variable; *y* is the sum of all observations; *x* is the variable; obs[*k*] is the sum of the *s*1 in the observed categorization given the *k*th categorical boundary; top[k] is the sum of the *s1* in the top categorization given the *k*th categorical boundary; and btm[*k*] is the sum of the *s*1 in the bottom categorization given the *k*th categorical boundary, in which the bottom categorization is defined by Kim^5^ as follows: “Suppose that all categorical boundaries in hierarchical stratification are fixed and observations are permutable. Bottom categorization means a condition in which observations are arranged in descending order in each category leading to descending order across all categories.”

### Real data set

A data set for a real rice population was used to compare the HH-CCDF with the *F* test and HA-coefficient algorithm. The population size is 278. The size of the marker set is 1617, in which each marker has a score of −1, 0, or 1 representing a single chromosome bin. A trait of interest was thousand grain weight (KGW). This data set was previously introduced by Xu^7^. The phenotypic observations consist of negative and positive values. The HA-coefficient algorithm requires only positive values for phenotypic observations, so the phenotypic observations were normalized into values ranging between 0 and 1 using the following equation:

(5) $$\begin{array}{l} z_{i}=\frac{x_{i}-\min\left(x\right)}{\max\left(x\right)-\min\left(x\right)}\\ i=\;1,\;2,\;3,\ldots ,a \end{array}$$

where $z_{i}$ is the normalized *i*th value which ranges between 0 and 1; $x_{i}$ is the *i*th value; min(*x*) is the minimum value; and max(*x*) is the maximum value.

The HH-CCDF and *F* test produce *P* values, so their results can be visualized into Manhattan plots by calculating −log_10_ (*P* values), whereas the HA-coefficients can be directly visualized into a plot. Comparing between results obtained by the 3 methods was conducted by overlapping 2 resulting plots at a time in the same panel, for which values resulting from each single method were normalized into values ranging between 0 and 1 using equation 5 for the purpose of overcoming the different scales resulting from different methods.

### R scripts

All computations were conducted using R.^8^ The R scripts are freely available at https://github.com/bongsongkim/HH-CCDF.

## Results

### Application of the HH-CCDF

Given Table 1, the phenotypic average for each marker score is as follows:

$$\begin{array}{l} {\bar{X}}_{2}=\frac{\left(116+108+108+97+91+86\right)}{6}=101\\ {\bar{X}}_{1}=\frac{\left(140+122+114+112+105+102+94+84\right)}{8}=109.125\\ {\bar{X}}_{0}=\frac{\left(105+101+99+98+95+76\right)}{6}=95.667 \end{array}$$

where ${\bar{X}}_{2}$ is the phenotypic average for marker score 2; ${\bar{X}}_{1}$ is the phenotypic average for marker score 1; and ${\bar{X}}_{0}$ is the phenotypic average for marker score 0.

Because ${\bar{X}}_{0}<{\bar{X}}_{2}<{\bar{X}}_{1}$, the marker scores can be sorted in the following order: 0, 2, 1. Therefore, the top categorization and observed categorization can be arranged as shown in Table 2.

**Table 2.** Table displaying the top and observed categorizations from Table 1.

Top categorization	0	0	0	0	0	0	1	1	1	1	1	1	1	1	2	2	2	2	2	2
Observed categorization	0	1	2	2	1	0	2	0	0	0	1	1	0	2	2	1	1	2	1	1
Phenotypic observations	86	91	97	108	108	116	84	94	102	105	112	114	122	140	76	95	98	99	101	105

**Figure 1.**
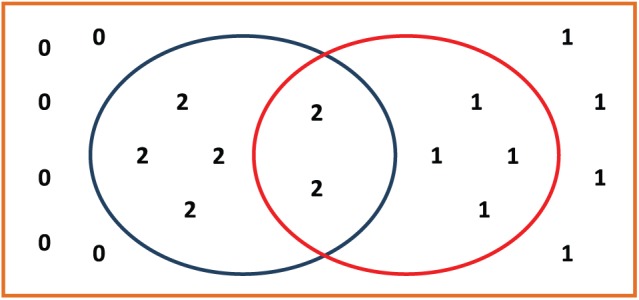
Diagram illustrating the relationship between the *s1* in the top categorization and the *s1* in the observed categorization at boundary 1, in which orange rectangle represents the whole population set; blue oval represents the *s1* in the top categorization at boundary 1; and red oval represents the *s1* in the observed categorization at boundary 1.

**Figure 2.**
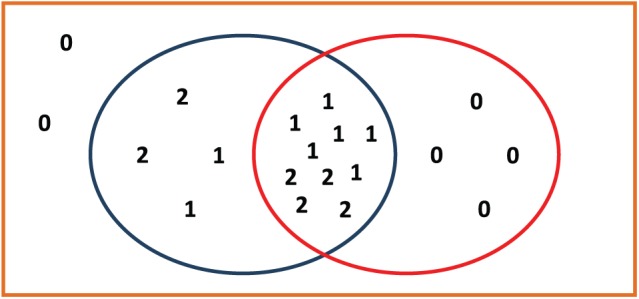
Diagram illustrating the relationship between the *s1* in the top categorization and the *s1* in the observed categorization at boundary 2, in which orange rectangle represents the whole population set; blue oval represents the *s1* in the top categorization at boundary 2; and red oval represents the *s1* in the observed categorization at boundary 2.

Table 2 is divided into 3 sections by the marker scores in the top categorization. Accordingly, 2 categorical boundaries exist, and a binary categorization can be set at each categorical boundary. Let us designate the boundary between marker scores 1 and 2 in the top categorization as boundary 1 and the boundary between marker scores 0 and 1 in the top categorization as boundary 2. Figures 1 and 2 show the relationships between the *s1* in the top categorization and the *s1* in the observed categorization at boundaries 1 and 2, respectively.

Let us collect the input values from Table 2 for the HH-CCDF. At boundary 1, *N* = 20 as the population size; *x* = 1 as the boundary number; $K_{1}$ = $d_{1}$ = 6 as the size of the *s1* at boundary 1; and $k_{1}$ = 2 as the size of the intersection between the *s*1 in the top categorization and the *s*1 in the observed categorization (see Figure 1). At boundary 2, *N* = 20; *x* = 2; $K_{2}$ = $d_{2}$ = 14; and $k_{2}$ = 10 (see Figure 2). All of the above values are summarized in Table 3.

**Table 3.** Summary of input values from Table 2 for the HH-CCDF.

Boundary	*N*	dx$d_{x}$	kx$K_{x}$	kx$k_{x}$
*x* = 1	20	6	6	2
*x* = 2	20	14	14	10

By applying the values in Table 3 to the HH-CCDF (equation 2), the *P* value can be calculated as follows:

$$P\;\;value=\sqrt{\left(1-\sum_{k_{1}=0}^{2}\frac{\left(\begin{array}{c} 6\\ k_{1} \end{array}\right)\left(\begin{array}{c} 14\\ 6-k_{1} \end{array}\right)}{\left(\begin{array}{c} 20\\ 6 \end{array}\right)}\right)\;\;\;\left(1-\sum_{k_{2}=0}^{10}\frac{\left(\begin{array}{c} 14\\ k_{2} \end{array}\right)\left(\begin{array}{c} 6\\ 14-k_{2} \end{array}\right)}{\left(\begin{array}{c} 20\\ 14 \end{array}\right)}\right)}=0.059$$

### Application of the HH-CCDF to the simulated data set

**Figure 3.**
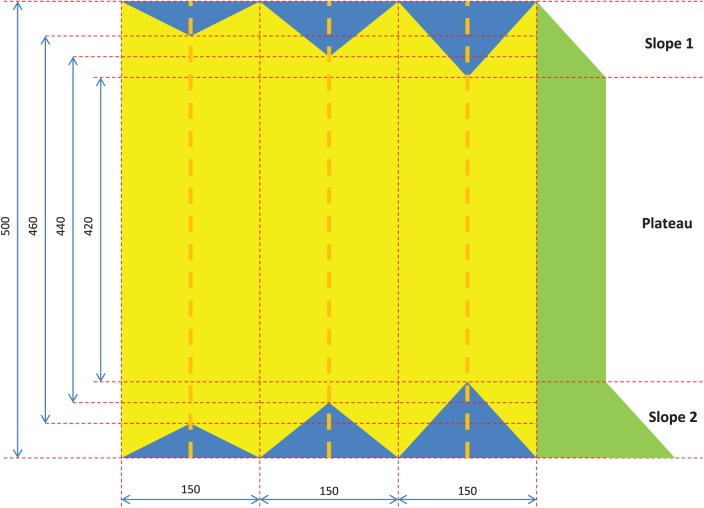
Diagram of the simulated data set in which the blue triangles on the top edge are full of 0s; the blue triangles on the bottom edge are full of 2s; the yellow zone is randomly filled with 0, 1, or 2; and the green area is a vector for phenotypic observations. The triangles on the top and bottom edges are symmetrical. Each dashed line in orange represents a column corresponding to the tips of each pair of symmetrical triangles.

**Figure 4.**
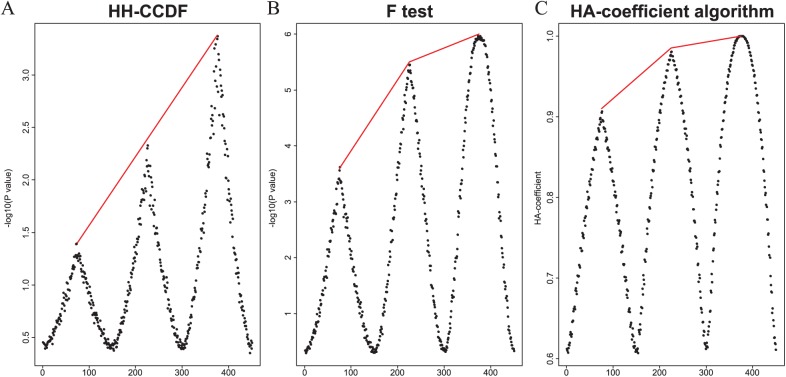
Plots obtained by applying the HH-CCDF (A), *F* test (B), and HA-coefficient algorithm (C) to Figure 3.

Figure 3 shows a diagram illustrating the simulated data set. The applied methods were the HH-CCDF, *F* test, and HA-coefficient algorithm. The resulting plots obtained by averaging the results from 100 iterative simulations are shown in Figure 4. At each plot, the left peak is shortest, and the right peak is tallest. The line connecting the 3 peaks is angled in the results obtained by the *F* test (Figure 4B) and HA-coefficient algorithm (Figure 4C). Meanwhile, the line connecting the 3 peaks obtained by the HH-CCDF (Figure 4A) is nearly straight.

### Application of the HH-CCDF to the rice data set

**Figure 5.**
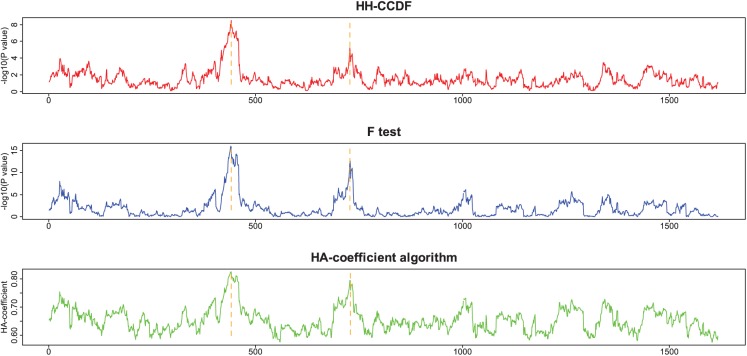
Plots obtained by applying the HH-CCDF (red), *F* test (blue), and HA-coefficient algorithm (green) to the rice data. Each plot has a different scale. In each plot, the orange-dashed lines are marked at 441 and 728, which are the first and second spots around which significant marker-phenotype associations are enriched, respectively.

**Figure 6.**
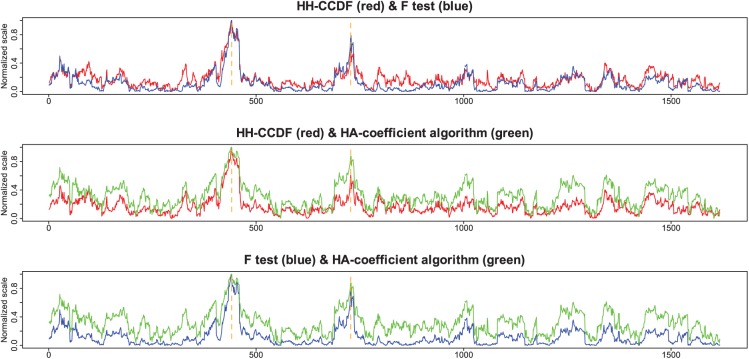
Pairwise comparisons between different plots. In each panel, 2 different plots are normalized between 0 and 1; the HH-CCDF, *F* test, and HA-coefficient algorithm are plotted in red, blue, and green, respectively; and the orange-dashed lines are marked at 441 and 728, which are the first and second spots around which significant marker-phenotype associations are enriched, respectively.

Figure 5 shows results obtained by applying the HH-CCDF, *F* test, and HA-coefficient algorithm to the rice data in which a trait of interest is the KGW. The plot of each method has a different scale, which makes comparisons between the 3 resulting plots difficult. To compare the 3 resulting plots in the same scale, each plot was normalized between 0 and 1; then, 2 different plots were compared at a time in the same panel (Figure 6). From all plots, the first and second spots around which significant marker-phenotype associations are enriched are found at 441 and 728, respectively. This suggests that the 3 methods are reliable in capturing significant marker-phenotype associations. Each plot as a whole shows a unique shape.

## Discussion

The HH-CCDF, *F* test, and HA-coefficient algorithm are applied to the simulated data set (Figure 3) for the purpose of comparing the ability to reveal the magnitude of gene signals. Figure 4 shows that a pair of symmetrical top and bottom triangles in Figure 3 collectively form a single peak by the HH-CCDF, *F* test, and HA-coefficient algorithm. In each column of Figure 3, the yellow section has nearly identical phenotypic averages for all marker scores, and the symmetrical blue sections have significantly different phenotypic averages. This means that the yellow section does not contribute to strengthening the marker-phenotype association, whereas the symmetrical blue sections significantly contribute. Because the total length of the symmetrical blue sections in each column is proportional to the marker-phenotype association, the blue sections can be regarded as visual representations of the gene signals. Therefore, the triangle heights increasing from left to right at regular intervals depict gene signals intensifying from left to right at regular intervals. Figures 4B and 4C show that lines connecting the 3 peaks obtained by the *F* test and HA-coefficient algorithm are angled. On the other hand, Figure 4A shows that the heights of 3 peaks obtained by the HH-CCDF increase from left to right at regular intervals. This regularity indicates that the HH-CCDF reveals the magnitude of gene signals in a robust manner, owing to the benefits of the count-based operation in measuring the distances between categories. When applied to the rice data set, the first and second spots around which marker-phenotype associations are enriched coincide across the 3 methods (see Figures 5 and 6). This suggests that the 3 methods consistently respond to significant marker-phenotype associations. Differences between results obtained by the HH-CCDF algorithm and the other methods are related to the fact that the HH-CCDF mitigates the impact of the imperfect proportionality between the gene-signal variable and phenotypic variable.

## Conclusion

Through examining the HH-CCDF, *F* test, and HA-coefficient algorithm with the simulated and rice data sets, it was identified that highly significant marker-phenotype associations were coincidently captured by the 3 methods. However, the simulation demonstrated that only the HH-CCDF properly revealed the magnitude of gene signals. This finding supports that the HH-CCDF is more robust than the other methods and may be able to provide us with possibilities to identify useful phenotype-associated markers which are undetectable by the other methods.

## Supplemental Material

Supplement_material_-_R_Code – Supplemental material for How to Reveal Magnitude of Gene Signals: Hierarchical Hypergeometric Complementary Cumulative Distribution Function Click here for additional data file. Supplemental material, Supplement_material_-_R_Code for How to Reveal Magnitude of Gene Signals: Hierarchical Hypergeometric Complementary Cumulative Distribution Function by Bongsong Kim in Evolutionary Bioinformatics
